# Totally laparoscopic associating simultaneous bile duct and portal vein ligation for planned hepatectomy for primary liver cancer: a case report

**DOI:** 10.1186/s13256-023-03859-4

**Published:** 2023-05-05

**Authors:** Wang Jinghan, Ao Jianyang, Ma Wencong, Liu Chen

**Affiliations:** 1 Department of Hepatobiliary and Pancreatic Surgery, Shanghai East Hospital, Tongji University School of Medicine, Shanghai, 200120 China; 2 Department of Biliary Tract Surgery I, Third Affiliated Hospital of Second Military Medical University, Shanghai, 200120 China

**Keywords:** Primary liver cancer, Portal vein, Bile duct, Ligation, Case report

## Abstract

**Background:**

Some patients with liver cancer lose the chance to have surgical treatment due to insufficient future remnant liver. To address this problem, individual or occlusion of both the portal vein and the bile duct was used to achieve quick hypertrophy. This is the first study reported in which simultaneous ligation of the portal vein and the bile duct was applied as the first step of planned hepatectomy of primary liver cancer.

**Case presentation:**

Here we report a case of a 38-year-old Asian male patient with hepatocellular carcinoma with tumor thrombus in the right anterior branch of the portal vein. Right hemihepatectomy can be curative, but patients face a high risk of liver failure because of the small volume of the remaining left liver lobe. Hence we developed a two-step liver resection strategy in which the patient underwent laparoscopic simultaneous bile duct and portal vein ligation of the right hepatic lobe prior to right hemihepatectomy under laparoscopy. Using this procedure, we achieved fast hypertrophy of the left liver lobe and successfully reversed the primary unresectability.

**Conclusion:**

This case report demonstrates that simultaneous bile duct and portal vein ligation may be a feasible option for those patients with liver cancer who cannot get surgical treatment due to insufficient future remnant liver.

## Background

Hepatectomy is the main method for the treatment of benign and malignant hepatobiliary diseases. However, frequently the volume of the future liver remnant is not sufficient, which limits the volume of liver resection and considerably decreases the chance for surgical treatment for some patients. Planned two-stage hepatectomy offers a possibility to resolve this problem [1]. Its principle objective is to achieve short-term atrophy of the resected hepatic tissue, while retaining hepatic hyperplasia. Currently, the first most commonly used step is portal vein ligation under laparoscopy or open surgery and selective portal vein embolization. Associating liver partition and portal vein ligation (ALPPS) has also become a recognized effective and feasible option, but the benefits of its application remain controversial [2]. Currently, the most frequently used method for promoting liver hyperplasia is to embolize the branch of the portal vein. However, animal experiments showed that ligating the bile ducts promoted rapid atrophy of the ipsilateral liver [3]. Other animal studies also revealed that the simultaneous ligation of the portal vein and the ipsilateral bile duct induces quick ipsilateral liver atrophy. Contralateral liver hyperplasia is a safe and feasible method.

In this study, the patient was a male patient with hepatocellular carcinoma with tumor thrombus in the right anterior branch of the portal vein. Right hepatectomy was planned, but the volume of the right liver lobe was too large, while the volume of the left liver lobe was small. Right hemihepatectomy can be curative, but patients face a higher risk of liver failure. Hence, we developed a planned two-step liver resection strategy. However, portal vein embolization (PVE) was not suitable in this case because a tumor thrombus was detected in the right branch of the portal vein. In the first step, we performed laparoscopic ligation of the right branch of the portal vein while ligating the right hepatic bile duct. The second step was laparoscopic right hepatectomy. Clinically, this method has been used less frequently, and thus no sufficient clinical experience is available. Here, we discuss the safety and feasibility of planned two-step hepatectomy for portal vein and bile duct ligation in patients with liver cancer.

## Case presentation

**Fig. 1:**
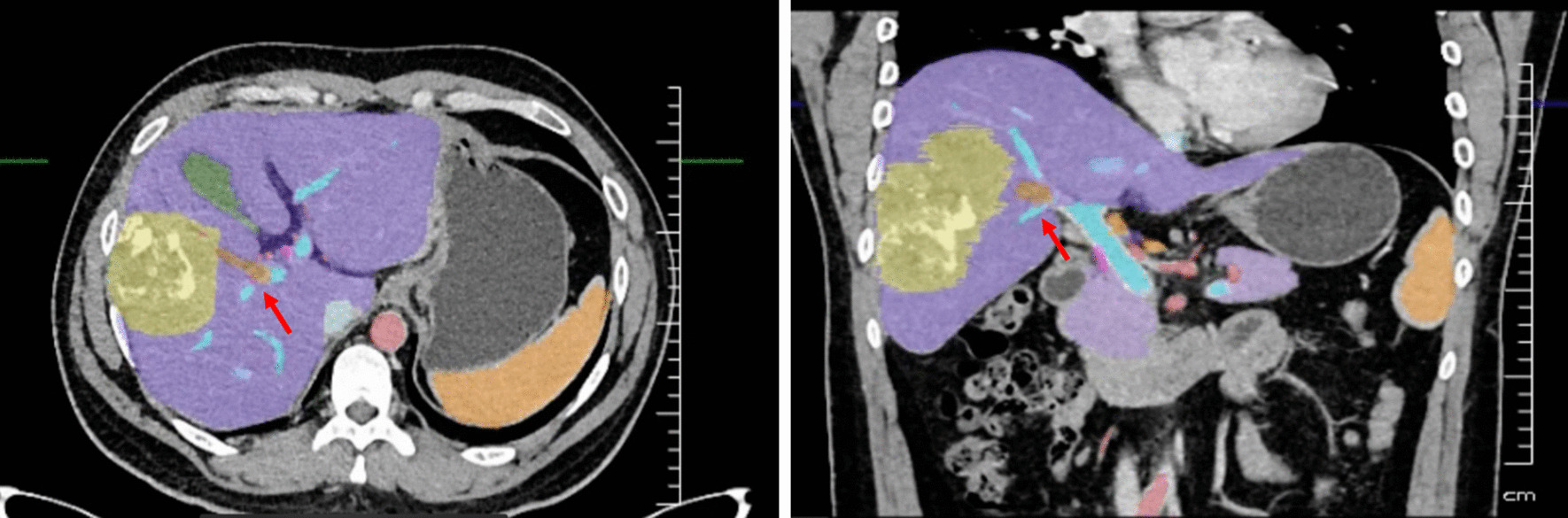
Preoperative Computed Tomography scan of liver, a right liver space-occupying lesion was found. The lesion was considered primary liver cancer with embolus of portal vein right anterior branch (red arrow)

A 38-year-old Asian male patient was diagnosed with right hepatocellular carcinoma accompanied with a right anterior portal vein tumor thrombus (Fig. 1). The patient received transcatheter arterial chemoembolization (TACE) at the local hospital before visiting our hospital. Blood tests suggested that his liver function was almost normal, with HBsAg^+^, anti-HBe^+^, and anti-HBc^+^. The serum levels of alpha-fetoprotein (AFP) and carbohydrate antigen (CA) 19–9 were 1200 ng/ mL and 14 U/mL, respectively. Theoretically, anatomic and right hepatectomy could achieve a radical curative effect. However, computed tomography (CT) data showed that the volume of the right liver lobe was large, whereas the volume of the future liver remnant was too small, which increased the risk of postoperative liver failure.

We further performed liver volume measurements and three-dimensional reconstruction of the liver. The total liver volume (TLV) was 2009.47 mL, and the volume of the left liver lobe, the future liver remnant volume (FLRV), was 621 mL (Fig. 3). FLRV was smaller than the physiological liver volume required for the patient [standard liver volume (SLV), 823.54 mL].

To achieve sufficient hypertrophy of the left liver lobe, we adopted a planned two-stage right hemihepatectomy strategy. The patient and his family did not agree with performing ALPPS, whereas PVE was not suitable due to the portal vein tumor thrombus. Thus, concurrent ligation of the right branch of the portal vein and the right bile duct was adopted. Informed consent was obtained from the patient and his parents prior to the surgical procedure. All surgical procedures were approved by the Ethics Committee of Third Affiliated Hospital of Second Military Medical University (Shanghai, China).

After all the preoperative preparations were performed and informed consent was obtained, the first step was to investigate the specific local status of the abdominal cavity and the tumor under laparoscopy. After the abdominal cavity and the tumor were detected, the gallbladder was removed, and the portal vein bifurcation and the right branch were carefully separated. Next, we pulled the right branch of the portal vein with 4–0 silk thread, and pinched the right branch of the portal vein with two ham-lock clamps near the confluence. During the ligation procedure, we tried to reach a maximally close proximity to the confluence to avoid embolization in the right anterior branch. Further, the right bile duct was ligated in the same way. Almost no bleeding occurred during the operation. Postoperatively, the liver function of the patient was significantly abnormal. During the first 3 days after the operation, transaminase levels sharply elevated. The leukocyte count also increased slightly, but there was no fever. We performed symptomatic treatments such as liver protection and prophylactic antibiotics, and closely monitored for changes in the vital signs. The patient’s general condition improved on the fifth day after surgery.

**Fig. 2:**
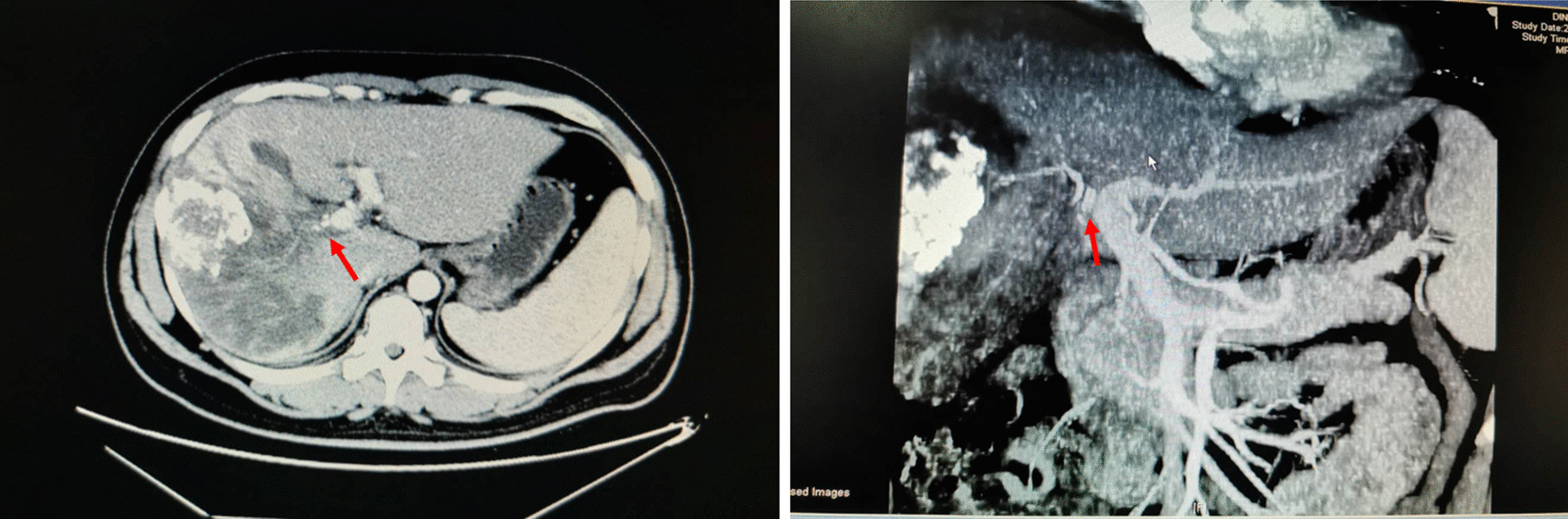
One week after the first postoperative operation, Computed Tomography scan of the liver showed that the right portal vein branch was occluded (red arrow). Moreover, part of the right liver presents a significantly low density, considering liver tissue necrosis

**Fig. 3:**
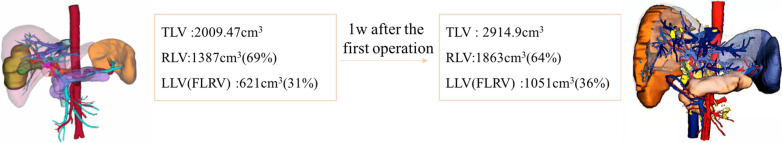
Three-dimensional reconstruction of the liver.* TLV* total liver volume,* RLV* right liver volume,* LLV* left liver volume,* FLRV* future liver remnant volume

Reevaluation was performed 1 week post-first-stage surgery. Contrast enhanced CT of the abdomen and liver volume measurement was conducted again (Fig. 2). The volume of FLRV was 1051.56 mL and of TLV was 2914.9 mL (Fig. 3). The TLV and FLRV values had almost doubled. Data showed that the volume rate of the left hepatic lobe decreased to 36.08%, but its volume increased. FLRV was significantly larger than the standard liver volume of the patient, and thus we considered surgery to be safe. The second step of the laparoscopic right hemihepatectomy was performed on the following day. The blood loss was 350 mL, and the duration was 360 minutes. No complication was observed in the intraoperative and postoperative course. The patient recovered very smoothly and was discharged from the hospital 1 week postoperatively. Histological examination showed hepatocellular carcinoma associated with microvascular damage. We recommend that patients return to the hospital 1 month after surgery to receive one-time preventive transcatheter arterial chemoembolization (TACE). The patient is still alive; he has been tumor-free and in good health for more than 6 months postoperatively.

## Discussion

Hepatectomy is the most important way to treat primary liver cancer. However, many patients are evaluated as unable to tolerate the procedure due to an insufficient future remnant liver (FRL). A large number of methods have been developed to promote FRL hypertrophy of and atrophy of the diseased liver.

It is well known that ligation or embolization of the portal vein of the unilateral lobe could induce atrophy of the unilateral hepatic lobe and hypertrophy of the future remnant liver. Portal vein embolization (PVE), a procedure devised by Makuuchi and Kinoshita, has been performed widely as a presurgical treatment of patients undergoing extended hepatectomy to minimize postoperative liver dysfunction [4]. PVE is currently our preferred method, especially in the treatment of primary liver cancer, hilar cholangiocarcinoma. However, patients who do not meet the PVE indications, especially those with a tumor thrombus in the trunk or the branch of the portal vein, can only choose to undergo portal vein ligation. On the other hand, PVL has been clinically used for a longer time. Honjo *et al*. applied this technique clinically to a case of liver metastasis in 1961 [5, 6]. Since then, portal vein ligation has been successfully used in many cases and has been recognized by physicians worldwide. It is noteworthy that Belghiti reported a planned two-step, totally surgical approach to clear all primary and metastatic disease [7]. In the first step, the primary tumor and all left-sided liver metastases are resected using straightforward resection techniques. Simultaneously, right portal vein ligation (RPVL) is performed to induce hypertrophy in the left lobe, which has been cleared of all detectable disease. After 4–8 weeks and hypertrophy of the disease-free FLR, a second step, consisting of a right or extended right hepatectomy, is planned to completely clear the remaining right-sided liver metastases.

Unilateral bile duct occlusion can promote ipsilateral liver atrophy and contralateral liver hyperplasia, which has been confirmed not only in animal models [3] but also in clinical cases [8]. Ligation of bile duct and portal vein simultaneously caused ipsilateral atrophy and contralateral hyperplasia of animal liver faster than only PVL. Naoki applied simultaneous ligation of the bile duct and portal vein in two-stage hepatectomy for treatment of multiple bilobar liver metastases from cecal cancer [8]. They first performed a right hemicolectomy and a segment-3 resection combined with microwave coagulation therapy of the metastatic tumors situated in the left hepatic lobe, together with simultaneous ligation of the right portal vein and right bile duct. Second, extended right hepatic lobectomy was performed.

## Conclusion

In the present case, we initially applied simultaneous ligation of the bile duct and the portal vein in the surgical treatment of primary liver cancer and successfully reversed the primary unresectability. No postoperative biliary tract infection was observed in our patient. The successful therapeutic outcome in this case should focus clinical attention on the possibility that, in some cases, simultaneous bile duct and portal vein ligation may be a feasible option, but the indications for the application of this approach must be well defined and evaluated. The main limitation of this procedure is the lack of sufficient cases reported in the literature to verify its reliability and establish and assess the risks for subsequent complications.

## Data Availability

All data generated or analyzed during this study are included in this published article.

## Abbreviations

PVE: Portal vein embolization
TACE: Transcatheter arterial chemoembolization
ALPPS: Associating liver partition and portal vein ligation
